# Research progress on the relationship between leaf senescence and quality, yield and stress resistance in horticultural plants

**DOI:** 10.3389/fpls.2022.1044500

**Published:** 2022-10-24

**Authors:** Wenxue Zhao, Huayuan Zhao, Huasen Wang, Yong He

**Affiliations:** ^1^ Collaborative Innovation Center for Efficient and Green Production of Agriculture in Mountainous Areas of Zhejiang Province, College of Horticultural Science, Zhejiang Agriculture and Forest University, Lin'an, Hangzhou, China; ^2^ Bashan Management Area of the Management Committee for Taishan Historic and Scenic Area in Tai’an City, Tai’an, China

**Keywords:** leaf senescence, yield, quality, stress resistance, post-harvest storage

## Abstract

Leaf senescence, the final stage of leaf development, is one of the adaptive mechanisms formed by plants over a long period of evolution. Leaf senescence is accompanied by various changes in cell structure, physiological metabolism, and gene expressions. This process is controlled by a variety of internal and external factors. Meanwhile, the genes and plant hormones involved in leaf aging affect the quality, yield and stress resistance in horticultural plants. Leaf senescence mediated by plant hormones affected plant quality at both pre-harvest and post-harvest stages. Exogenous plant growth regulators or plant hormone inhibitors has been applied to delay leaf senescence. Modification of related gene expression by over-expression or antisense inhibition could delay or accelerate leaf senescence, and thus influence quality. Environmental factors such as light, temperature and water status also trigger or delay leaf senescence. Delaying leaf senescence could increase chloroplast lifespan and photosynthesis and thus improve source strength, leading to enhanced yield. Accelerating leaf senescence promotes nutrient redistribution from old leaves into young leaves, and may raise yield under certain circumstances. Many genes and transcriptional factors involved in leaf senescence are associated with responses to abiotic and biotic stresses. WRKY transcriptional factors play a vital role in this process and they could interact with JA signalling. This review summarized how genes, plant hormones and environmental factors affect the quality, yield. Besides, the regulation of leaf senescence holds great promise to improving the resistance to plant biotic and abiotic stresses.

## 1 Introduction

Leaf senescence is the final stage of leaf development. It is the adaptive mechanism formed by plants in their long-term evolutionary process. The most evident morphological indicator of leaf senescence is the change in leaf color from green to yellow. Accompanied by the decrease of chlorophyll content, the protein content and net photosynthesis rate decline during leaf senescence. In general, leaf senescence is mainly controlled by the developmental age (Song et al., 2014). However, leaf senescence may be triggered by environmental clues. It has been suggested that ‘programmed cell death’ (PCD) in plants occurs at a specific stage of intrinsic senescence and becomes irreversible after leaf yellowing reaches a “point of no return” (Sobieszczuk et al., 2018). As the last step of leaf development, leaf senescence affects plant productivity and quality, either positively or negatively.

With the improvement of living standard and yield required for the increasing population, more attention is drawn to pre-harvest management and post-harvest management of plant cultivation. Once horticultural plants are harvested, the leaves have certain similarities with the aging caused by development. The plant leaves are like unique windows (Jan et al., 2018), revealing various changes of cells, tissues, and organs during aging. For instance, Reactive oxygen species (ROS) are accumulated during leaf senescence, contributing to the degradation of chlorophyll. On the contrast, increase Carbohydrate-related metabolism Plasma-Activated Water (PAW) enhanced the resistance to "chilling injury" and delayed leaf senescence in spinach, PAW treatment may contribute to the preservation of valuable nutrients (Uhlig et al., 2021). Plants usually redistribute nutrients from senescent leaves to other organs (Woo et al., 2019), and this strategy could be beneficial for the survive of plants under abiotic stresses, indicating low and high temperatures, drought stress. In addition, plant hormones may delay or advance leaf aging, Zhang et al. elaborated the role of salicylate and ROS in leaf senescence and plant immunity (Zhang et al., 2020). Furthermore, some genes are involved in leaf senescence. These genes are related to the degradation of proteins, nucleic acid and chlorophyll degradation and they are identified mainly by using mutants related to the aging process.

**Figure 1:**
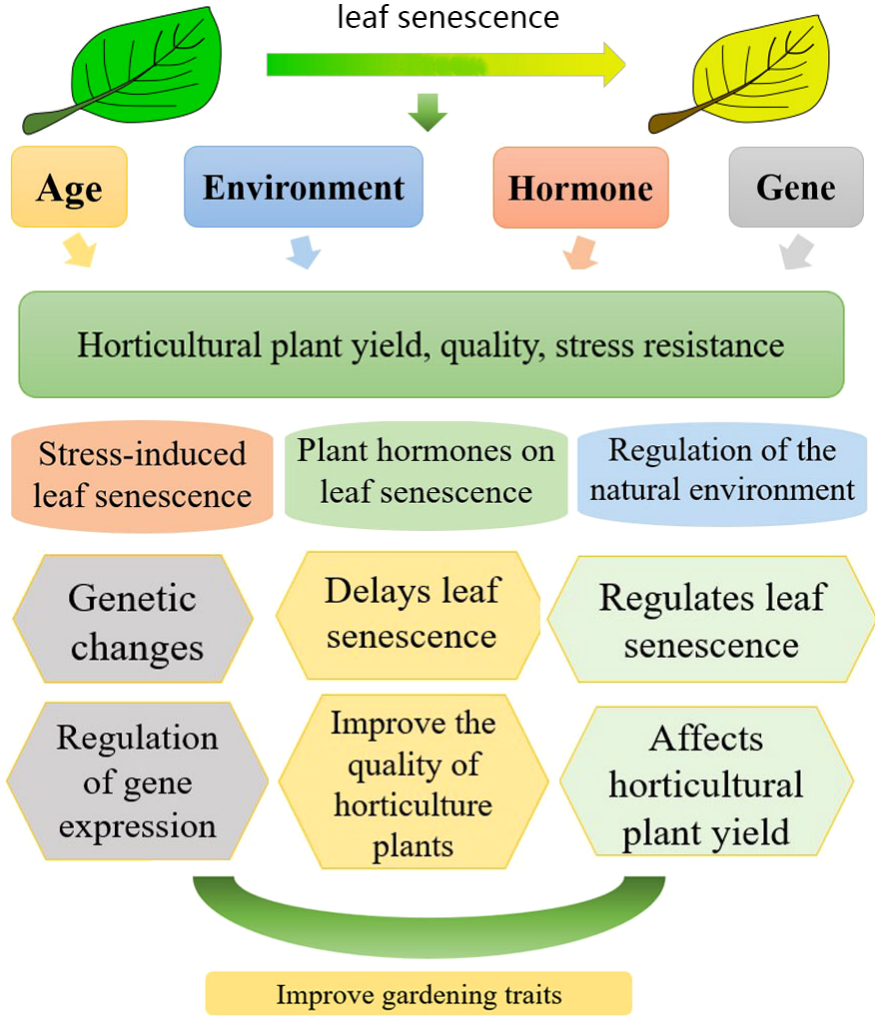
Relationship between leaf aging and the yield, quality and stress resistance in horticultural plants (leaf senescence of horticultural plants is subject to the influence of hormones, genes, exogenous hormones, and growth age control; aging has a certain effect on the yield, quality, and stress resistance of horticultural plants.).

In this review, we summarized the relationship between leaf senescence and the quality, productivity, and stress resistance in horticultural plants (**Figure 1**). We focused on the molecular mechanism of leaf senescence. This review aimed to gain insights into the regulation of leaf senescence in horticultural industry. This provides a basis for maximizing yield and quality and enhancing stress resistance.

## 2 Leaf senescence and horticultural plant qualities

### 2.1 Plant hormones regulate leaf senescence and affect the quality of horticultural plants

**Figure 2:**
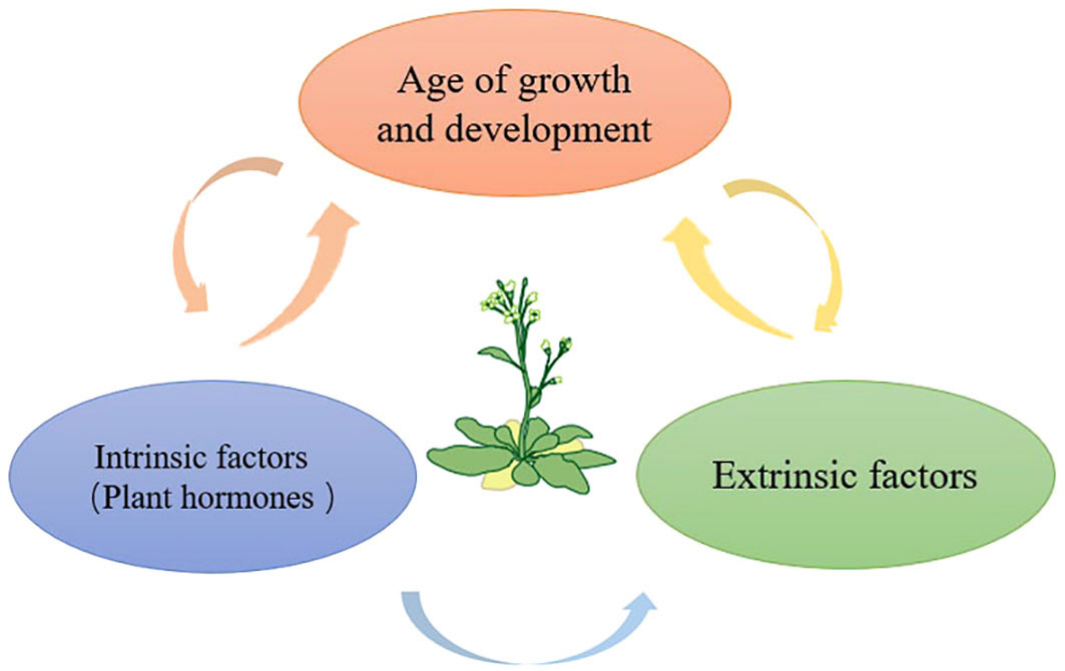
Leaf senescence is the final stage of leaf development, and leaf aging is regulated by a variety of factors, such as strict developmental age, exogenous factors and endogenous factors, thereby regulating the whole process of plant growth and development.

Plant hormones affect most stages of the plant life cycle, including leaf senescence. Meanwhile, plant hormones are key players in the quality formation of horticultural plants. Thus modification of phytohormones could affect both leaf senescence and product quality (**Figure 2**).

#### 2.1.1 Plant hormones regulate leaf senescence to affect pre-harvest quality

Exogenous hormones can delay the aging of pre-harvest leaves, increase photosynthesis, promote the accumulation of assimilates, and thus improve quality. For example, 10~80 mg/L 1-Naphthaleneacetic acid (NAA) and Gibberellin A_3_ (GA_3_) have significant effects on preventing A4 seedless lychee from falling fruit before harvest and improving the quality of fruit. Among them, treatment at 40 mg/L had the best effect on reducing the pre-harvest fruit drop rate. Treatment with GA_3_ at a concentration of 20 mg/L increased the single fruit weight of lychee (Yang et al., 2015). Cut flower roses sprayed with 1-Methylcyclopropene (1-MCP) before harvest have a storage period of up to 26 days. The 1-MCP treatment significantly extends the life of the bottle insert for 0 or 6 days for refrigerated flowers, extending them by 27.1% and 42.6%, respectively (Huang et al., 2017). During the growth process of thick-skinned melon, the leaves usually appear after fruit setting, and leaf aging causes their yellowing. Thus, photosynthesis rate cannot maintained, resulting in decreased yield and fruit quality. Exogenous 6-BA (Zhu, 2015) and exogenous calcium nitrate (Zhang et al., 2014) affected the aging of thick-skinned melon leaves and thus influenced their quality. Zhu et al. observed the soluble solid content and vitamin C content of T2 treated fruits were 12.3% and 11.3% higher than those of the control. (Zhu, 2015). Comprehensive previous studies have revealed that exogenous hormones slowed down the aging degree of leaves to a certain extent, prolonged photosynthesis, promoted the accumulation of assimilates, and improved the quality of horticultural plants.

#### 2.1.2 Plant hormones regulate leaf senescence to affect post-harvest quality

For foliage plants and leafy vegetables, the status of leaves is an important attribute of product quality. For instance, the leafy vegetables should not be wilt, and have green color (Hassan and Mahfouz, 2012; Miret et al., 2018). Ethylene(ET) are considered to be involved in post-harvest process and leaf senescence (Tomotsugu, 2018). The release of ET during post-harvest promoted the over-accumulation of reactive oxygen species (ROS), leading to the yellowing of cabbage leaves (Yu and Xi, 1997). Low temperatures can inhibit ET production, thereby slowing down the yellowing of leaves and inhibiting chlorophyll degradation (Yu and Xi, 1997). Exogenous ET promoted the leaf yellowing and senescence and degradation of chlorophyll in fresh-cut chrysanthemums (Liu et al., 2022b). Treatment with 1-MCP at a concentration of 0.5 μl/L reduced respiration in the *Glebionis coronaria*, alleviated the decline in protein content, and extended shelf life. However, low concentration of 1-MCP (< 0.5 μl/L) did not affect shelf life of the *Glebionis coronaria*. (Ma, 2006).Gibberellic acid (GA) interacts with ET *via* DELLA proteins (Achard et al., 2006), and thus affect leaf senescence. During the storage process of Chinese cabbage, exogenous GA inhibited the expression of aging-related genes, delayed leaf aging and maintained a high chlorophyll content (Xiao et al., 2019). Pre-harvest spray of 20 mg·L^-1^GA_3_ significantly reduced the rot rate of fruits during storage period, increased the accumulation of malondialdehyde (MDA), and delayed the degradation of vitamin C, thereby inhibiting the aging of Gongci fruit (Ma et al., 2009). Moreover, GA_3_-treated cabbage maintained a high maximum quantum yield (Fv/Fm) and total chlorophyll content (Fan et al., 2018).

Abscisic acid (ABA) is a key player in stomatal closure, and thus influence the water loss during post-harvest. Manipulation of ABA levels has an important effect on postharvest quality of leafy vegetables. Miret observed ABA can inhibit leaf senescence during storage, thereby prolonging the shelf life of vegetables (Miret et al., 2017). ABA reactive protein *MdBT2* interacted with *MdbHLH93* to induce ubiquitination and degradation of *MdbHLH93* protein, thereby delaying leaf senescence in apple (An et al., 2019). The ABA pretreatment significantly reduced the brown detergentilence of the ‘Yellow Crown’ pear, improved the ability of POD, APX and peel free radical to varying degrees, and reduced the MDA content (Liu et al., 2017). ABA treatment is used to inhibit the aging of leafy vegetables, improve the postharvest quality of storage at room temperature, and delay the loss of sensory metabolites.

Cytokinins bear upon leaf senescence in horticultural plants. An increase in cytokinin level is thought to block the aging process of leaves associated with age and stress, thereby preserving the photosynthetic properties of the leaves (Lira et al., 2017). Chen et al. discovered that after Forchlorfenuron (CPPU) treatment, the degradation rate of soluble proteins after 3 d of storage at room temperature was significantly slower than that of the control cabbage. CPPU can inhibit the activity of cabbage heart protein hydrolases and slow down yellowing, thereby improving the quality of postharvest gardening plants (Chen et al., 2001). 6-BA can delay the aging of yellow flowers of cauliflower (Gao, 1998).

Based on the analysis of previous studies, exogenous hormones can delay leaf aging, thereby improving the photosynthesis of plant leaves, which has an important effect on postharvest storage. This property extends the shelf life and promotes the postharvest quality of horticultural plants, which helps to increase in their market value.

### 2.2 Leaf senescence under genetic regulation affects the quality of horticultural plants

In modern horticultural production, genetic modification could become a key strategy for improving biomass and nutritional quality. Genes that change in expression levels during leaf senescence are called leaf senescence- associated genes (SGAs). In previous studies, a large number of SAGs have been isolated in rice (**Table 1**). These senescent genes are involved in macromolecular degradation, nutrient redistribution, transcriptional regulation, stress defense, etc. (Leng et al., 2017; Woo et al., 2019). In tomatoes, promoters SGA12/SGA13 come into play, flowering earlier and increasing chlorophyll content, thereby increasing yield (Swartzberg et al., 2006). Antisense inhibition of *GIGANTEA*(*BoGI*) in kale (*Brassica oleracea* var. *sabellica*) delayed both leaf senscence and flowering (Thiruvengadam et al., 2015).

**Table 1:** Promoter/Transcription factor regulates the effects of leaf senescence on horticultural plants (n.d. = not determined).

Plant	Promoter/Transcription factor	Chlorophyll	Biomass/yield	References
Tomato	AGPaseS1	n.d.	n.d.	Luo et al. (2005)
Tomato	SAG12/SAG13	Advanced flowering	Fruit weight increases	Swartzberg et al. (2006)
Canola	AtMYB32	Chlorophyll levels are high	Production increases	Kant et al. (2015)
eggplant	SAG12	Higher than wild type	Yield increases	Xiao et al. (2017)
*Rosa hybrida*	RhGA20ox1	n.d.	n.d.	Lü et al. (2014)
*Brassica napus L.*	SAG12	Decreased chlorophyll content of old leaves and nitrogen deficiency	n.d.	Gombert (2006)
cucumber	SAG12	Chlorophyll increases	Production increased	Liu et al. (2022a)
Camellia oleifera Abel.	SAG12	Delay chlorophyll degradation	Yield increased	Yang et al. (2020)
Tomato	SAG12/SAG13	n.d.	n.d.	Swartzberg et al. (2011)
Gerbera	SAG12/SAG13	n.d.	Extend lifespan	Huang et al. (2021)
Betula platyphylla	BpEIN3.1	n.d.	Yield increased	Song et al. (2022)
Chinese flowering cabbage	BrNAC087	n.d.	n.d.	Fan et al. (2021)
Tomato	SlERF.F5	The accumulation of chlorophyll content is reduced	n.d.	Chen et al. (2022)
Tomato	SlNAP2	Production increases	Production increases	Ma et al. (2018)
*Brassica napus L.*	*BnaNAM*	n.d.	n.d.	Wang et al. (2022)
Chinese flowering cabbage	*BrNAC041*	n.d.	n.d.	Fan et al. (2020)
*Brassica napus L.*	*BnaWGR1*	n.d.	n.d.	Yang et al. (2018)
Chinese flowering cabbage	*BrTCP21*	n.d.	n.d.	Xiao et al. (2019)

Leaf aging affects the quality of apple fruit, Wang et al. cloned the *MhYTP1* and *MhYTP2* genes, and found that their expressions were positively correlated with plant leaf senescence, suggesting that these genes can be indicator of plant senescence (Wang et al., 2017).Tomato transcription factor SlWRKY37 can regulate dark leaf senescence, which can reduce tomato sensitivity to external aging signals, thus providing target genes for delayed leaf yellowing (Wang et al., 2022). Tomato *MYC2*, as a key factor in leaf senescence, transduces jasmonic acid (JA) signaling and induces JA-mediated senescence in tomato leaves by accelerating chlorophyll degradation and inhibiting carbon fixation (Ding et al., 2022). *BnaNTL1* is a membrane-bound NAC (NAM, ATAF and CUC) transcription factor (TF) in rape (Yan et al., 2021) and it is expressed primarily in senescent leaves and can actively regulate ROS production. By regulating key genes, *BnaNTL1* can promote the fruit ripening period and postharvest storage, regulate the ripening time, and extend shelf life. Through the regulation of leaf aging genes, the quality of horticultural plants is improved to a certain extent and the shelf life is extended.

### 2.3 Leaf senescence and horticultural plant quality under environmental regulation

Environmental stresses such as drought, waterlogging, high-temperature, low-temperature, soil salinity stresses, are important factors restricting plant productivity. Environmental stress regulates leaf aging, affecting both quality and yield in horticultural plants.

#### 2.3.1 Temperature

In general, low temperature during post-harvest inhibits the leaf respiration and delays leaf senescence. Low temperature provides a way for spinach post-harvest preservation storage by delaying the decline of antioxidant enzyme activity and leaf aging (Tian et al., 2014). Under low-temperature storage, especially at 0° conditions, it can maintain the high nutrient content of slow down leaf aging, prolong shelf life, and solve the problem of rapid yellowing of leaves (An et al., 2020). In *Arabidopsis thaliana*, low temperature inhibited the phyB nucleus output, thereby inhibiting the transcriptional activity of ET signaling media and delaying leaf senescence (Lee et al., 2022). On the contrast, high temperature acceleated leaf aging, decreasedthe soluble protein content of strawberries, which directly affected the photosynthetic ability of strawberries (Yang et al., 2012).

#### 2.3.2 Light

Both light intensity and light quality affect the leaf senescence and plant quality in horticultural plants. Extremely low light intensity such as darkness accelerates leaf aging and affects grain and oil yield in sunflower (Dosio et al., 2020). During low light intensity, carbon fixation efficiency determines leaf senescence; however, plant photoreceptor pigment A has a regulatory effect on carbon fixation efficiency (Brower et al., 2012). High light intensity could induce light inhibition and damage. Shading net is a routine measure in horticulture production. Under the color shading net, the quality of the grapes is better than those under black shading nets because the absence of high light intensity causes premature leaf failure, which improves the quality of grapes; the soluble solids are also affected differently under various shade nets, whereas unshashed grapes show thermal damage (Zha et al., 2022). Studies have shown that Supplemental lighting(SL) sources affect the physiology and productivity of plants. Supplemental lighting (SL) within the canopy increases the overall tomato yield and maintains the nutrient content of plants (Gómez and Mitchell, 2016). Besides, light quality has a certain influence on the growth of rhododendrons. Exposure to short period of ultraviolet radiation per day improved water utilization inside plants, thereby delaying the aging of tomato leaves, favoring plants to cope with drought conditions, and playing a key role in water-saving irrigation in agriculture (Mannucci et al., 2022). RBL( red and blue lights) can increase the synthesis of photosynthetic pigments and improve antioxidant enzyme activity. This process delays leaf aging and ensures plant growth. (Wang,Y. et al.,2017)

#### 2.3.3 Moisture

Maximizing leaf lifespan is the key to tea quality; drought and cold have a combined effect on leaf senescence and tea quality (Zheng et al., 2016). Zhao et al. (2016) reported that PYL9 and leaf senescence play an important role in coping with drought stress. PYL9 inhibits drought by limiting water loss during respiration, and the combination of ABA receptor PYL9 and other transcription factors accelerates leaf senescence, thereby improving drought tolerance and survival in plants. The upregulation of SAG12 gene and downregulation of *Cab* gene reflects the transition of sinks/sources in cabbage-type rape leaves, which are accurate physiological indicators of leaf aging and can effectively identify the degree of nitrogen deficiency in leaves (Gombert, 2006).

The environment is essential for the adaptability and productivity of plants. Leaf aging is an important stage of development for productivity and quality. Natural environmental factors such as light, temperature, and moisture all regulate leaf aging, which affects the quality of horticultural plants.

## 3 Leaf senescence and horticultural plant yield

### 3.1 Increasing horticultural plant yield by increasing chloroplast lifespan

Leaf senescence is caused by the gradual loss of green pigment and the degradation of chlorophyll (Gan and Hörtensteiner, 2013). Thomas hypothesized that extending the maximum photosynthetic period delays the aging process, thereby increasing yield (Thomas and Stoddart, 1980). Photosynthesis is the basis of plant growth and material accumulation and important for nutrient accumulation, quality formation and sustainable development of production capacity. Manipulating leaf aging-related genes is an effective strategy to improve pulp yield (Lira et al., 2017), that is, by delaying leaf aging to prolong the photosynthetic characteristics of leaves, thereby enhancing source activity. Zhao et al. (2022) revealed the changes in photosynthetic ability of grape leaves during development and aging and analyzed the nutrient absorption of leaves during the growing season; their results showed significance for improving the yield of horticultural plants. ROS has been identified as a dependent signal to mediate leaf aging, analyzed the differences between dark-induced and natural aging, and proposed to increase plant yield by increasing the chloroplast lifespan (Bartoli et al., 2013; Weaver et al., 1998; Buchanan-Wollaston et al., 2005).

The shortage of assimilates in leaves eventually accelerates the maturation process of plants (Gan, 2003), strongly affecting plant productivity. The most evident manifestation of leaf senescence is the breakdown of chlorophyll into non-green derivatives, which causes the yellowing of leaves (Mayta et al., 2019). As the keys to nutrient cycling, chloroplast degradation and Rubisco play important roles, with protein degradation starting in chloroplasts (Feller et al., 2008). Given that chloroplasts contain most of the nutrients, they are the main source during plant carbon and nitrogen cycles. The disintegration of photosynthetic organs is a developmental stage in the aging process. ROS production is likely to be caused by changes in the electronic pathway and photosynthetic organs. Chloroplasts are the most important site of ROS production (Laloi and Havaux, 2015). The subcellular localization of *OsPLS4*, which is found in chloroplasts, chloroplast development and biosynthesis of rice cuticle waxes are affected by *OsPLS4* mutations, decreased photosynthetic rate, and premature leaf aging (Zhou et al., 2020). Cytokinin slows down leaf senescence and prevents chlorophyll degradation and destruction (Talla et al., 2016). Delaying leaf aging and maintaining the green color of leaves has various meanings, including provision of more carbon and promoted root growth; thus, the onset of aging promotes the increase in plant yield.

### 3.2 Raising yield *via* delaying of leaf senescence

**Figure 3:**
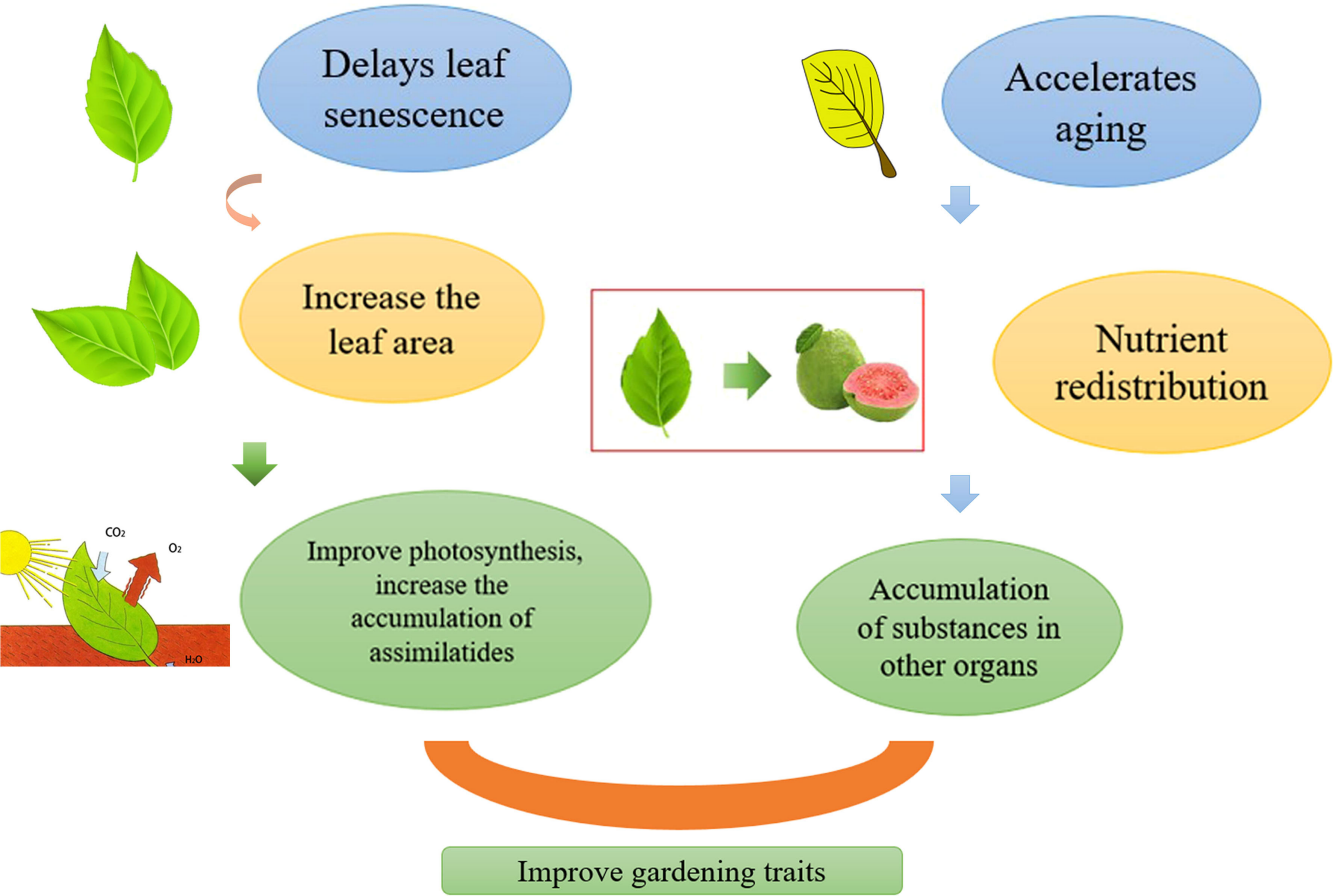
Regulation of leaf aging affects horticultural plant yield, delays leaf aging, increases leaf area, thereby improving photosynthesis and increasing the accumulation of assimilates. Accelerates leaf aging, redistributes nutrients, and promotes the accumulation of substances in other organs. Both pathways can increase yields.

Improving the photosynthetic efficiency is the basis for increasing yields. Delaying the aging of plant leaves, which increases the leaf area to a certain extent, is conducive to plant photosynthesis, thus increasing the accumulation of assimilates (**Figure 3**).

The study revealed that *pSARK-IPT* transgenic plants can increase CO2 fixation under drought conditions and delay leaf aging, thereby increasing tolerance to extreme drought. Moreover, the yield of genetically modified plants has increased significantly (Blumwald et al., 2009). For the peach tree, the aging of roots and leaves is delayed, and the nutrient storage level is increased, thereby increasing the fruit yield (Zhang et al., 2021). Shading delays the aging of peony leaves, thereby increasing photosynthetic capacity. The development of seeds and accumulation of nutrients are prolonged, thereby increasing the yield (Han et al., 2020). Improving nitrogen utilization can delay the aging of rapeseed leaves. Varieties with high nitrogen use can adapt to low light conditions during flowering. Studies have also shown a positive correlation between nitrogen use and delayed leaf senescence (Schulte auf’m Erley et al., 2007). Precise regulation of leaf aging enables plants to respond quickly to environmental signals. In apples, *MdAB15* inhibits the transcriptional activity of downstream target genes *MdNYE1* and *MdNYC1*. This condition slows down the aging of ABA-induced leaves (An et al., 2021). DCPTA is sprayed on the foliar surface of sugar beet to enhance leaf development and photosynthetic productivity. Rubisco activity is increased to a certain extent, demonstrating an increase in plant net photosynthesis. Delayed leaf aging increases net carbon assimilation of leaves (Keithly et al., 1990). In the late stage of growth and development, especially in the period of yield formation, the delayed leaf aging and maintenance of the leaf area are keys to improving the accumulation of dry matter. In summary, delaying the onset of leaf aging can improve photosynthesis, which in turn increases horticultural plant yields.

### 3.3 Improving yield through nutrient redistribution mediated by accelerated leaf senescence

As leaves begin to age, the materials they have accumulated during the growth phase are converted into nutrients that can be exported and supplied to other tissues and organs (Woo et al., 2019). Aging is accompanied by the reuse of nutrients, which can increase plant yield to a certain extent (**Figure 3**). When a plant is subjected to adversity stress, the leaves age early, allowing the plant to adapt better to the environment. For several annual plants, nutrients are transferred to the fruit and grain as the leaves age. A study showed that C2H2-type zinc finger *MdZAT10* can accelerate the aging of apple leaves (Yang et al., 2021). In Arabidopsis, knocking out *HDA15* can accelerate leaf aging and promote plant flowering (Huang et al., 2022). In safflower, *CtCP1* promotes the hydrolysis of the CtbHLH41 protein, accelerating the transcriptional activity of related genes and leaf senescence (Hong et al., 2022). A study revealed that *MdHHO3* promotes early leaf aging under nitrate deficiency, which proves that the lack of nitrates can cause aging of apple leaves (Wen et al., 2022). The sink-source relationship affects leaf aging, and the large source of the sink or the large source of the sink will lead to abnormal leaf maturity. Both the sink and the source are important factors that determine yield. If plant vegetative growth is too vigorous, reproductive growth is restricted, resulting in reduced yield and quality. Therefore, to ensure the coordination of the relationship between the sink and the source, we can obtain high-yield and high-quality horticultural crops. Reproductive growth also promotes leaf senescence to a certain extent. A competition for nutrition exists between vegetative and reproductive organs. In summary, accelerating leaf senescence can increase yield to a certain extent.

### 3.4 Genes regulate leaf senescence and horticultural plant yield

Genetic modification can delay leaf aging, maintain photosynthesis for a long time, and sustain leaf activity, thereby increasing yield. According to Kant et al., the IPT gene encodes cytokinin synthesis to delay the aging of rapeseed leaves under flooding conditions. During long periods, transgenic rapeseed maintains the high chlorophyll levels, which improves crop yield under water stress conditions (Kant et al., 2015). At the genetic level, plant responses to stresses differ significantly from aging procedures and are associated with accelerated aging process (Guo and Gan, 2011). Leaf senescence caused by environmental stress or intrinsic inheritance can reduce photosynthesis, lead to premature cell death, and cause the supply of anabolites to decrease before flowering, thus negatively regulating the final grain weight (Gregersen et al., 2008). In numerous annual plants, such as monocotyledonous plants, aging is usually controlled by reproductive structures. Mature leaves are regulated by environmental and other organ physiological signals that trigger reversible events, which eventually lead leaves at to a “point of no return”, which is a sign of the onset of irreversible aging (Lim et al., 2007). Genes, such as the NAC transcription factor *S1NAP2* that controls the aging of tomato leaves. This prolongs the photosynthesis time of the leaves, which increases the fruit yield and sugar content of tomatoes (Ma et al., 2018). In cucumbers, transient expression of the MT and MT synthetic gene *CsASMT* inhibits leaf senescence under dark conditions. MT delays leaf aging, reduces the rate of decline in chlorophyll, and promotes photosynthesis. This condition speeds up the growth of cucumbers and increases yield (Liu et al., 2022a). In Chinese cabbage leaf senescence, ET reactive factor (ERF) works in conjunction with JA. The transcription factor *BrERF72* is a nucleus-localizing protein that activates the expression of JA biosynthetic genes by directly targeting its promoter (Tan et al., 2018). In summary, leaf senescence is not controlled by a single gene, but by a polygenic regulatory system. Predecessors revealed that the aging gene, which regulates leaf aging at the molecular level, provides a basis for anti-aging breeding of horticultural plants and increasing yields.

## 4 Leaf senescence and plant stress resistance

### 4.1 Leaf senescence and disease resistance of horticultural plants

For plants that are frequently exposed to adverse conditions, biological and abiotic stresses are inevitable during the growing season (Gregersen et al., 2013). Leaf senescence is an important component of plant development and an adaptation strategy for plants to cope with stress. It can also reduce pathogen transmission. Zhang et al. observed that powdery mildew and propabenzole (PBZ)-activated ethylene (ET) signal transduction, upregulates the expressions of *CSEIN3* and *CsCCGs* and *CsRBOHs*, leads to leaf senescence, and improves the plant’s resistance to powdery. An invading pathogen causes a “suicide” hypersensitivity reaction in leaves, which triggers a cascade of immune responses and a slow green loss phenomenon, which is indicative of the rapid aging process of cells (Zhang et al., 2022). Wang et al. have also shown that cucumber anthrax and downy mildew resistance is directly regulated by NYE1/SGR1 (Wang et al., 2019).

The improvement of leaf disease resistance is an important factor for the increase in plant yield. Plant leaf aging reduces the invasion of pathogens to a certain extent. Numerous genes that control leaf aging are also involved in plant stress resistance. In eggplant, P-SAG12-IPT overexpression plays a role in delayed leaf aging while enhancing tolerance to drought and cold; the activity of ROS-scavenging enzymes is higher than that of wild-type eggplant, and growth rate and yield are relatively improved (Xiao et al., 2018).

WRKY18 works with WRKY40 and WRKY60 and can modulate disease resistance. WRKY18, WRKY40, WRKY60 three mutants and double mutants WRKY18, WRKY40 and WRKY18, WRKY60 are significantly more resistant to lilac. In addition, in terms of resistance to powdery mildew fungi, two mutants, WRKY18, are more resistant to WRKY40 (Xu et al., 2006). WRKY70 expression has a diametrically opposite effect on the resistance of Arabidopsis necrotrophic bacteria and biotrophic bacteria (Li et al., 2006). Mutant *nore1-1*was found in EMS mutagenesis of *Arabidopsis thaliana*; it is associated with leaf senescence, where the mutant activates the defense system, accelerates leaf senescence, and improves the plant’s disease resistance (Lee et al., 2016). Salicylic acid (SA) and reactive oxygen species (ROS) can regulate leaf senescence and defend against pathogen invasion. The WRKY55 transcription factor accelerates leaf senescence and improves resistance to Pseudomonas syringum by modulating these two factors (Wang et al., 2020). In rice, the overexpressed *OsNBL1* interacts with the localized protein *OsClpP6*, delays leaf senescence, contains a higher chlorophyll content than wild-type plant leaves, and is critical for salt and disease resistance (Zhao et al., 2021). Leaf aging plays a key role in disease resistance. Regulating leaf aging is conducive to horticultural plants to cope with different diseases, and to a certain extent, the resistance of plants is improved.

### 4.2 Leaf senescence and stress resistance of horticultural plants

Regulation of the aging process in leaves is a part of the adaptation of plants to different environmental conditions, especially drought stress. Usually, numerous transgenic plants exhibit matching drought resistance and green leaf traits. Darkness can induce leaf senescence, and shading is an important way to improve plant performance (Brower et al., 2012). Field crops will benefit from shady and dark conditions, resulting in nutrient remobilization after leaf aging, which is conducive to the photosynthesis of plants. Under the induction of dark environment and JA, the aging rate of plant leaves significantly improves. Specifically, SlWRKY37 plays a positive role in regulating leaf aging in tomatoes. Wang et al. revealed the function of SlWRKY37 in leaf aging, and delayed leaf yellowing by reducing the sensitivity to external aging signals through a target gene (Wang, Z. et al. 2022). Sucrose phosphatase prevents plant leaf senescence under drought stress conditions (Sami et al., 2016). Cytokinin levels in roots are suppressed due to nutrient deficiencies, causing leaf senescence and allowing nutrient transport (Maillard et al., 2015; Havé et al., 2016). Delays the onset of leaf senescence, improves photosynthesis, and as nutrients are transferred from old leaves to young leaves, the antioxidant capacity of plants increases to resist stress and allow normal growth to return (Jan et al., 2018). WRKY transcription factors have an important influence on leaf senescence and environmental stress. Gu et al. isolated the gene GhWRKY91 from WRKY, and its transgenic plants showed a strong drought tolerance, delayed degree of leaf aging in an arid environment, enhanced gene expression associated with drought stress, and relatively weakened aging gene (Gu et al., 2019). Tea plants generally grow under cold and drought stress conditions, which affect their quality and leaf aging. Zheng et al. discovered that drought induction delays leaf aging, thus enhancing antioxidant capacity, prolonging photosynthesis, and revealing the relationship between quality, stress resistance, and leaf life (Zheng et al., 2016). Lee et al. identified the drought-responsive NAC transcription factor NTL4, which promotes the production of ROS in senescent leaves under drought stress and accelerates leaf aging; however, mutants lacking NTL4 show delayed leaf senescence and improved drought resistance (Lee et al., 2012). Accelerate leaf senescence and improve nutrient reuse, pepper leaves, after inoculation with CMV, accelerate senescence and improve resistance, and the difference between CMV-inoculated resistant peppers and sensitive peppers and changes in their photosynthesis was studied (Stefanov et al., 2011). Hu et al. reviewed the relationship between JA-mediated leaf senescence and tolerance to cold stress and observed that JA treatment can increase the expression of senescent genes (Hu et al., 2017). The drought tolerance of sorghum after flowering is tightly bound to the greening of leaves, thereby increasing biomass (Harris et al., 2019). Overexpressed *SlERF5* in transgenic tomatoes can maintain leaf moisture status and chlorophyll concentrations, giving plants strong salt stress and drought tolerance; however, studies have shown the minimal influence of these findings on production (Pan et al., 2012). The short period of ultraviolet radiation per day has a certain effect on improving water utilization inside the plant, thereby delaying the aging of Tom tomato leaves, favoring plants to cope with drought conditions, and playing a key role in water-saving irrigation in agriculture (Mannucci et al., 2022). The use of ABA has a slight effect on the yield composition and vegetative growth of grapes, but it can lead to aging, shedding, and dormancy of grape leaves, which increases freezing tolerance (Li and Dami, 2015). Delaying the aging of plant leaves can improve the stress resistance of plants to a certain extent. After the aging of the leaves is slowed down, photosynthesis is enhanced, which is conducive to plants coping with various stressful environments.

## 5 Conclusions and perspectives

This paper reviews the relationship between leaf aging and horticultural plant quality, yield, and stress resistance. Through the quality analysis of horticultural plants before and after harvest, most of the reports revealed a certain relationship with leaf aging. Delayed leaf aging plays an important role in prolonging the shelf life of vegetable fruits and is conducive to occupying a huge market potential. We summarized the different roles of various plant hormones which are an important part of regulating plant quality, in the storage of horticultural plants. Through the analysis of gene regulation, that of the WRKY family, NAC family, etc., the potential mechanism of regulating leaf aging, which is the key to maintaining optimal productivity and improving the survival of offspring, can be studied to optimize the productivity of horticultural plants. In addition, we analyzed the possible effects of various environmental conditions on the quality of horticultural plants, and concluded that light, temperature, and moisture all played a pivotal role. Through the summary analysis of leaf senescence and plant yield, delaying the onset of leaf senescence and improves photosynthesis, thereby promoting the accumulation of assimilates to increase yield. Meanwhile, accelerating the aging rate of leaves, improves the reuse of nutrients and promotes the accumulation and maturation of fruit nutrients. Although photosynthesis and photoconjugates play important roles in leaf aging, the molecular mechanisms of aging induction need to be further studied. Controlling leaf aging has important implications for agricultural and horticultural production. Screening mutants with missing functions during leaf aging is beneficial to unravel the mystery of the molecular mechanism of leaf senescence. We believe that by delaying leaf aging, coupled with improved photosynthesis and tolerance to stress, it is possible for horticultural plants to achieve stable yields and yield potential. Leaf senescence is an important part of plant development, but also an adaptation strategy for plants to cope with stress, and the regulation of the aging process is an important method for plants to adapt to different environmental conditions. In summary, the relationship between aging and productivity, quality, and stress tolerance is complex. To achieve high yield and quality of horticultural crops, experts in different fields must work together.

## Author contributions

YH, WZ and HW conceived and designed the review. WZ wrote the manuscript, and YH proposed revisions to the manuscript. All authors contributed to the article and approved the submitted version.

## Funding

This study was supported by Lingyan Project of Science and Technology Department of Zhejiang Province (Grant No.2022C02051), the National Natural Science Foundation of China (Grant Nos.31872105, 31972221, 32002048), Ministry of Agriculture, and the National Key Research and Development Program of China (Nos.2018YFD1000800 and 2019YFD1000300)

## Conflict of interest

The authors declare that the research was conducted in the absence of any commercial or financial relationships that could be construed as a potential conflict of interest.

## Publisher’s note

All claims expressed in this article are solely those of the authors and do not necessarily represent those of their affiliated organizations, or those of the publisher, the editors and the reviewers. Any product that may be evaluated in this article, or claim that may be made by its manufacturer, is not guaranteed or endorsed by the publisher.
